# Broadband Solar Absorber and Thermal Emitter Based on Single-Layer Molybdenum Disulfide

**DOI:** 10.3390/molecules29184515

**Published:** 2024-09-23

**Authors:** Wanhai Liu, Fuyan Wu, Zao Yi, Yongjian Tang, Yougen Yi, Pinghui Wu, Qingdong Zeng

**Affiliations:** 1 School of Intelligent Manufacturing, Zhejiang Guangsha Vocational and Technical University of Construction, Jinhua 322100, China; wanh2006@126.com; 2 Joint Laboratory for Extreme Conditions Matter Properties, Southwest University of Science and Technology, Mianyang 621010, China; 15284169398@163.com (F.W.); tangyongjian2000@sina.com (Y.T.); 3 College of Physics and Electronics, Central South University, Changsha 410083, China; yougenyi@csu.edu.cn; 4 College of Physics & Information Engineering, Quanzhou Normal University, Quanzhou 362000, China; phwu@zju.edu.cn; 5 School of Physics and Electronic-Information Engineering, Hubei Engineering University, Xiaogan 432000, China; zengqingdong2005@163.com

**Keywords:** ultra-wideband absorption, two-dimensional materials, molybdenum disulfide, thermal emission

## Abstract

In recent years, solar energy has become popular because of its clean and renewable properties. Meanwhile, two-dimensional materials have become a new favorite in scientific research due to their unique physicochemical properties. Among them, monolayer molybdenum disulfide (MoS_2_), as an outstanding representative of transition metal sulfides, is a hot research topic after graphene. Therefore, we have conducted an in-depth theoretical study and design simulation using the finite-difference method in time domain (FDTD) for a solar absorber based on the two-dimensional material MoS_2_. In this paper, a broadband solar absorber and thermal emitter based on a single layer of molybdenum disulfide is designed. It is shown that the broadband absorption of the absorber is mainly due to the propagating plasma resonance on the metal surface of the patterned layer and the localized surface plasma resonance excited in the adjacent patterned air cavity. The research results show that the designed structure boasts an exceptional broadband performance, achieving an ultra-wide spectral range spanning 2040 nm, with an overall absorption efficiency exceeding 90%. Notably, it maintains an average absorption rate of 94.61% across its spectrum, and in a narrow bandwidth centered at 303 nm, it demonstrates a near-unity absorption rate, surpassing 99%, underscoring its remarkable absorptive capabilities. The weighted average absorption rate of the whole wavelength range (280 nm–2500 nm) at AM1.5 is above 95.03%, and even at the extreme temperature of up to 1500 K, its heat radiation efficiency is high. Furthermore, the solar absorber in question exhibits polarization insensitivity, ensuring its performance is not influenced by the orientation of incident light. These advantages can enable our absorber to be widely used in solar thermal photovoltaics and other fields and provide new ideas for broadband absorbers based on two-dimensional materials.

## 1. Introduction

In contemporary society, with the sharp increase in energy demand, the supply of traditional fossil energy has been struggling to meet the needs of sustainable development, prompting people to focus on a wider range of renewable energy fields [1,2,3]. Among them, solar energy, as an emerging and clean form of renewable energy, has attracted much attention. In order to cope with the challenge of energy shortage, researchers have carried out in-depth and extensive research on various types of clean energy, including solar energy [4,5,6]. However, despite extensive research on solar absorbers, there are still many drawbacks. For example, the narrow width of the absorption band, the low absorption intensity, and the complex structure limit the application of absorbers in solar photovoltaic and other fields. Therefore, it is important to explore a broadband absorber with good oblique incidence characteristics and polarization angle independence as well as high thermal radiation efficiency [7,8,9].

In addition, ultra-wideband absorbers constructed from refractory materials show significant promise for applications where thermophotovoltaic devices are frequently subjected to high-temperature extremes. These absorbers not only operate stably at high temperatures, but also maintain excellent absorption stability, which lays a solid technical foundation for efficient energy conversion in high-temperature environments. Therefore, an in-depth exploration and optimization of the design strategy and preparation technology of such absorbers is of great significance to accelerate the innovation and development of solar energy and wider clean energy technologies. Titanium metal is known for its unique physical properties, including core advantages such as high strength, excellent heat and corrosion resistance, superior ductility, and relatively low density [10]. As a member of the refractory metals, titanium has a high melting point of 1668 °C, displays excellent thermal stability, and exhibits good antimagnetization properties in strong magnetic field environments [11]. In absorber applications, titanium stands out not only for its excellent stability, but also for its cost-effectiveness compared to precious metals such as gold (Au) and silver (Ag). Of particular interest is the ability of titanium as a resonant material to excite a broader bandwidth response in the infrared spectral region, a property that offers the possibility of realizing highly efficient absorption in an ultra-broad band, thus greatly broadening its potential for a wide range of practical applications.

Over the past few years, two-dimensional materials have garnered significant attention within the scientific community, primarily due to their unparalleled physical and chemical characteristics, such as high electron mobility, excellent heat resistance, and chemical stability. Graphene [12,13], as a leader in the field of two-dimensional materials [14,15,16,17], has attracted extensive research interest for its unique physical properties. Its ultra-thin characteristics are particularly remarkable, with a thickness of only 0.34 nm, which is almost equal to the diameter of a single carbon atom. As an allotrope composed of carbon elements, in its monolayer state, the carbon atoms of graphene are closely combined with three adjacent carbon atoms by SP hybridization, forming a unique planar hexagonal honeycomb structure [18]. This structure endows graphene with excellent mechanical, electrical, and thermal properties, which makes it show great potential in scientific research and industrial applications. Many studies have confirmed that these excellent properties of graphene provide a broad prospect for its application in many fields [19,20,21,22]. Graphene is unique in its zero-band gap property, which enables electrons to transition from valence band to conduction band at a very low energy state. However, it is the energy band structure of graphene that has zero bandgap [23], so it has no adjustable semiconductor conductivity at all, which limits the possibility of its further development [24].

Therefore, in contrast to pristine graphene, transition metal dichalcogenides (TMDCs) are generally regarded as superior absorbing materials. This is due to the fact that graphene possesses a zero bandgap, necessitating thermal excitation of electrons for conductivity, a characteristic that significantly constrains its practical applicability. TMDCs exhibit a tunable bandgap spanning from 1 to 2 eV, offering significant advantages in the fabrication of absorbers. Key attributes that render TMDCs particularly suitable for the production of solar absorbers include their remarkable stability, controllable thickness, and high absorption efficiency. Their ultrathin (monolayer) nature endows them with a direct bandgap within the visible spectrum, resulting in exceptional absorption capabilities. Among two-dimensional materials, MoS_2_ stands out as a highly promising candidate for functional photonic devices due to its notable current cutoff ratio and tunable optoelectronic properties, as reported in [25,26]. Consequently, this study focuses on single-layer MoS_2_ as a representative example for our investigation into two-dimensional materials.

Molybdenum disulfide (MoS_2_), a transition metal sulfide, has received a lot of attention in recent years for its potential applications in optoelectronics. The unique properties of MoS_2_, especially its broadband light absorption capability in the visible to near-infrared region, make it an ideal material for solar absorbers [27]. Compared with other two-dimensional materials, MoS_2_ has a direct bandgap, which gives it a significant advantage in light absorption efficiency. In addition, the high electron mobility of MoS_2_ facilitates the rapid transport of electrons under light excitation, which improves the response speed and efficiency of optoelectronic devices. In terms of chemical stability, MoS_2_ is able to maintain its performance under a wide range of environmental conditions, which is essential for the fabrication of durable optoelectronic devices [28]. With the development of solution processing techniques, the preparation of MoS_2_ thin films has become more economical and scalable. Spin-coating techniques have been used to prepare homogeneous MoS_2_ films and characterize them by variable angle spectroscopic ellipsometry. In addition, by using the dimethylformamide/n-butylamine/2-aminoethanol solvent system, researchers have been able to synthesize wafer-scale MoS_2_ thin films with controllable thickness using the solution method. These advances not only improve the quality of MoS_2_ films, but also pave the way for their integration in solar cells, photodetectors, and other optoelectronic devices [29,30]. The bandgap of MoS_2_ can be tuned by chemical doping or strain engineering, enabling precise control of the absorption spectrum. This tunability provides great flexibility in designing optoelectronic devices with specific spectral responses. With the further understanding of MoS_2_ material properties and the continuous advancement of processing technologies, MoS_2_-based optoelectronic devices are expected to play an important role in the future of sustainable energy and advanced electronics.

Currently, the state of absorbers in this domain is marred by numerous limitations: some absorbers are too complicated to design and too bulky for actual manufacturing [31]. The working range of the absorber is too narrow, the absorption capacity is limited, and numerous state-of-the-art absorbers utilize costly precious metals, particularly gold and silver, resulting in significant production expenses. Additionally, there is ample scope for enhancing their thermal endurance to ensure optimal performance under various conditions. Thus, in this paper, we propose a cost-effective solar absorber featuring straightforward manufacturing processes, a broad absorption spectrum, and superior absorption efficiency within the solar radiation spectrum. The solar energy absorber demonstrates remarkable absorption capabilities across a broad spectral domain, particularly within the wavelength interval spanning from 280 to 2320 nanometers (2040 nm in total), and its absorption efficiency keeps above 90%, with an average absorption efficiency as high as 94.61%, which fully proves its high-efficiency light energy absorption capacity. Therefore, our absorber will provide some references for similar structures of other solar absorbers and has potential for a wide range of applications such as solar thermal photovoltaic systems. Our work also opens up new avenues for the application of two-dimensional materials in the field of optoelectronics and energy conversion.

## 2. Results and Discussion

**Figure 1:**
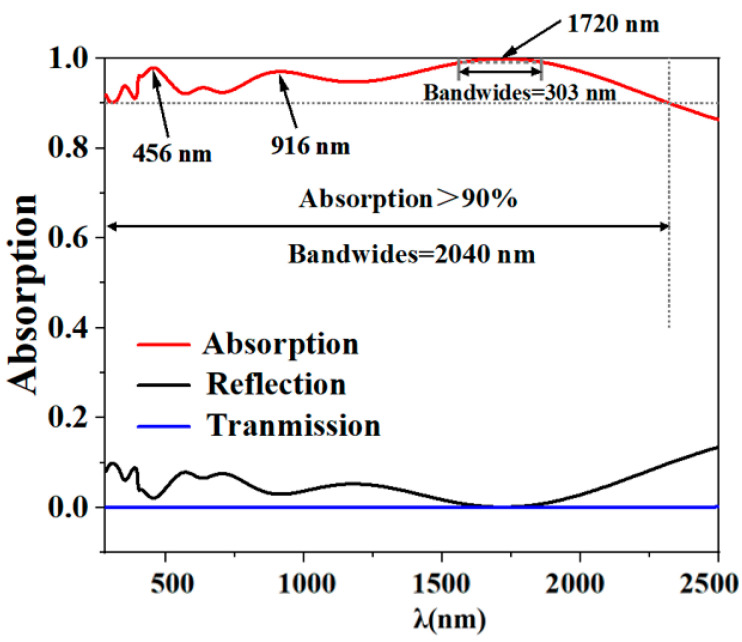
Depicts the spectral characteristics of absorption, reflectance, and transmittance, under plane incident light.

Figure 1 meticulously portrays the absorption characteristics of the structure through a comprehensive chart. Specifically, the red trace delineates the absorption efficiency, whereas the black trace signifies reflectivity, and the blue trace is indicative of transmittance. In view of the sufficient thickness of the substrate Ti, we observed that the transmittance was almost zero. Obviously, the solar energy absorber shows excellent absorption performance in a wide spectral range, particularly within the spectral range spanning from 280 to 2320 nanometers (a total of 2040 nm), within which the absorber maintains an absorption efficiency exceeding 90% with an average efficiency of 94.61%. Notably, it achieves a near-perfect absorption rate of 99% specifically at a wavelength of 303 nm, which fully proves its high-efficiency light energy absorption ability. Compared with other recent absorbents based on molybdenum disulfide, our absorbent has obvious advantages in bandwidth and average absorption rate, as shown in Table 1 [12,32,33,34,35,36,37].

In order to probe into the rationale behind the ultra-broadband and near-perfect absorption achieved in this paper, we chose three representative bands for the follow-up study of electric field distribution. These three bands are λ1 = 456 nm (in the visible region), λ2 = 916 nm (in the vicinity of the infrared spectral region) and λ3 = 1720 nm (also in the near-infrared region). Through this analysis, we expect to reveal the key factors and mechanisms to achieve efficient absorption.

A comprehensive comparison of the performance metrics of the absorber introduced in this study with those reported in prior research is presented in Table 1. The analysis shows that the perfect absorber designed by us has remarkable ultra-wide bandwidth characteristics, which obviously exceed other comparative absorbers, and its highest absorption rate is also better. In addition, although our solar energy absorber is not the highest in terms of average absorption efficiency, it still shows significant advantages in comprehensive performance of bandwidth and efficiency, which provides us with an ideal and practical choice.

### 2.1. The Influence of Different Structures on the Results

**Figure 2:**
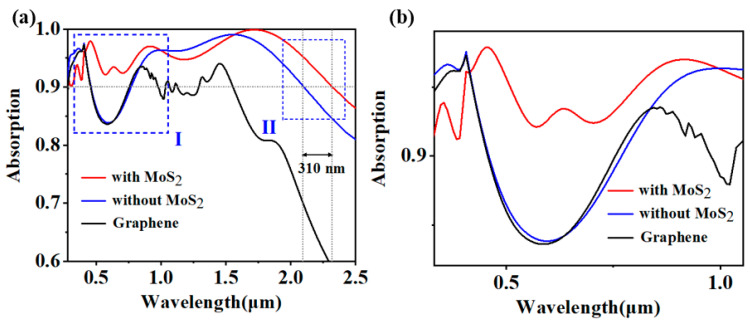
(**a**) Illustrative sketch outlining the enhancement in absorption for a monolayer MoS_2_ structure; (**b**) Magnified section of Region I for closer inspection.

Initially, we delve into the consequences of integrating a single-layer molybdenum disulfide within the architecture. For illustrative purposes, we have modeled the structure with and without a single layer of molybdenum disulphide and with the transformed 2D material being graphene. For example, the absorption of light by monolayer graphene is inherently low, about 2.3 per cent, due to its energy band structure [39]. Graphene is a zero-bandgap material with an electronic structure similar to a Dirac cone, which makes it less responsive to light in the visible and near-infrared bands, as shown in Figure 2a. As evident from Figure 2a, the incorporation of this monolayer yields two notable benefits: a significant broadening of the operational bandwidth and an enhancement in the absorption efficiency within the shorter wavelength region. Within Region I, the analysis reveals that the absorption rate of the nanostructures falls below 90% across a relatively extensive bandwidth, in the absence of a single layer of molybdenum disulfide. After the introduction of single-layer molybdenum disulfide, it can be seen that due to its high absorption rate in the near-ultraviolet band, the absorption efficiency within the shorter wavelength spectrum undergoes a substantial augmentation, surpassing the 90% threshold. Furthermore, Figure 2b, depicting a magnified section of Region II, evidences a marked expansion in the bandwidth where the absorption rate surpasses 90%. Specifically, the introduction of a single layer of MoS_2_ results in a broadening of this high-absorption region by approximately 310 nanometers. In a word, the introduction of single-layer molybdenum disulfide makes the Ti-SiO_2_ cuboid structure designed for superior absorption address the challenge of insufficient absorption in the short-wavelength region, thereby effectively broadening the operational bandwidth.

**Figure 3:**
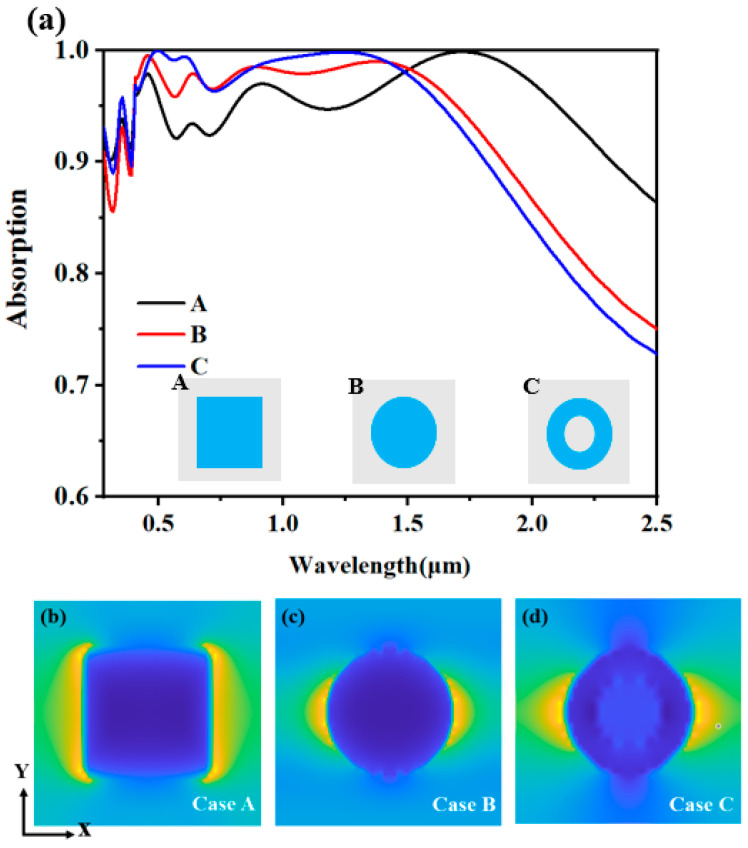
(**a**) is an absorption spectrum diagram of different micro-nano structures; (**b**–**d**) are the electric field intensities of different micro-nano structures in the XOY plane within a period.

In the following research, we deeply discuss the influence of different geometric patterns on the performance of the absorber. We replaced the original cuboid structure with a cylindrical structure (B) and a circular column structure (C), respectively, and calculated their absorption efficiency under the same lighting conditions. Through careful analysis of the absorption spectrum in Figure 3a, our analysis indicates that the rectangular parallelepiped configuration exhibits a marginally reduced absorption efficiency compared to both cylindrical and circular cylindrical structures, within the spectral range spanning from 400 nm in the visible light region to 1600 nm in the near-infrared, but it shows significant advantages in the band over 1600 nm. Its wider bandwidth and higher overall absorption rate of solar full spectrum grant the cuboid structure greater potential in wide-band light absorption applications. Based on the above analysis, we finally chose the cuboid structure as the micro-nano-structure of the absorber in order to achieve better performance. This selection not only considers the evaluation of the absorber’s efficacy within the visible light and near-infrared spectral bands, but also fully considers its comprehensive performance in a wider band.

In addition, from the electric field distribution diagram shown in Figure 3b–d, we can observe that the electric field is mainly concentrated on the geometric surface. This phenomenon is mainly attributed to the excitation of PSPs (surface plasmon) on the surface of the pattern layer [38,40,41]. PSPs are electromagnetic oscillations generated at the metal-medium interface; this capability allows for the conversion of light energy into thermal energy or alternative energy forms, thus achieving efficient light absorption [42,43,44]. Therefore, the intensified electric field distribution along the geometric surface underscores the pivotal role played by PSPs in facilitating the light absorption process.

### 2.2. Physical Mechanism Analysis of High Absorption and Wide Bandwidth of Structure

**Figure 4:**
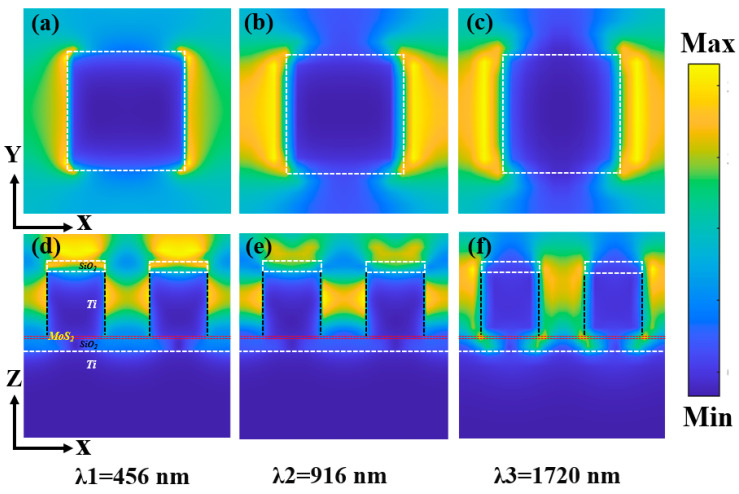
(**a**–**c**) are the electric field distributions of the XOY plane in one period at λ1, λ2, and λ3 wavelengths, respectively; (**d**–**f**) are the electric field distributions of two periodic XOZ planes at λ1, λ2, and λ3 wavelengths, respectively.

To gain deeper insight into the underlying physical mechanisms that contribute to the high absorption efficiency and extended bandwidth of the absorber, spanning from the visible light to the near-infrared region, we have conducted calculations pertaining to the electric field distributions at specific wavelengths: λ1 = 456 nm, λ2 = 916 nm, and λ3 = 1720 nm, and compared them by drawing. Among them, Figure 4a–c shows the electric field distribution in the XOY direction in the next period from λ1 to λ3, and Figure 4d–f shows the electric field distribution in the XOZ direction in the two periods from λ1 to λ3.

Integrating the analysis of the electric field distribution within both the X-Y and X-Z planes, as shown in Figure 4a–f, with the increase in resonance wavelength, optical coupling goes from the edge of the metal cuboid of the pattern layer to the air cavity formed by the adjacent pattern layers, and it is obvious that excitation of surface plasmon resonance occurs within the absorber [45,46]. When the wavelength is λ1 = 456 nm, observation of Figure 4a reveals that the electric field is predominantly concentrated on the metallic upper segment of the patterned layer. Therefore, the primary factor contributing to the perfect absorption observed at wavelength λ1 is the excitation of surface plasmon polaritons (PSPs) on the surface of the patterned layer.

Different from the excitation mechanism of PSPs, the excitation of LSPs does not need specific momentum-matching conditions [47,48,49]. The underlying reason for this is that the incident light’s wavelength far exceeds the characteristic dimensions of the metallic structures, and these nanostructures can be regarded as a kind of focused source point, which can produce diverse wave vector components, and then provide the necessary momentum matching for LSPs in the excitation structure. More specifically, the excitation intensity of local surface plasmon resonance (LSPs) is determined by the wavelength of the incoming light, the size of nanoparticles or nanostructures and the inherent characteristics of the materials [50]. Since the wavelength of incident light, λ2 = 916 nm, is much longer than the period of the absorber, 400 nm, LSPs can be generated in the metal of the pattern layer.

Illustrated in Figure 4b,e, a strong electric field appears in the adjacent pattern layer region. The electric field distribution shows that PSPs and LSPs are excited on the metal surface of the pattern layer and in the adjacent pattern air cavity, respectively; concurrently, the resonant wavelength of λ2 = 916 nm attains perfect absorption. At a wavelength of λ3 = 1720 nm, the electric field distribution in the XOY direction is similar to that when λ2 = 916 nm, as shown in Figure 4c, but different from 916 nm. An intense electric field accumulates within the dielectric spacer, localized at the interface between the cuboid structure and the MoS_2_ layer, and the electric field intensity of the top anti-reflection layer silicon dioxide is weakened. Therefore, the perfect absorption observed in the 1720 nm band stems from the excitation of localized surface plasmons (LSPs).

### 2.3. Weighted Average Absorption Rate and Radiation Performance Analysis

This paper delves into the absorption and radiation properties of the proposed structure, with the objective of evaluating its performance efficiency as both a solar absorber and a thermal emitter. In this process, we use the global spectral equation under the condition of AM 1.5 incident solar energy, which is specifically expressed as [51,52]:

(1) $$\eta_{A}=\frac{\int_{\lambda_{Min}}^{\lambda_{Max}}A\left(\omega \right)I_{AM1.5}\left(\omega \right)d\omega }{\int_{\lambda_{Min}}^{\lambda_{Max}}I_{AM1.5}\left(\omega \right)d\omega }\;\;$$

The equation of thermal emission efficiency (*η*_E_) is [53]:

(2) $$\eta_{E}=\frac{\int_{\lambda_{min}}^{\lambda_{max}}\varepsilon (\omega )\cdot I_{BE}(\omega ,T)d\omega }{\int_{\lambda_{min}}^{\lambda_{max}}I_{BE}(\omega ,T)d\omega }\;\;$$

Herein, *I_BE_*(*ω*, *T*) signifies the intensity of radiation emitted by an ideal blackbody at a given frequency *ω* and temperature *T*.

**Figure 5:**
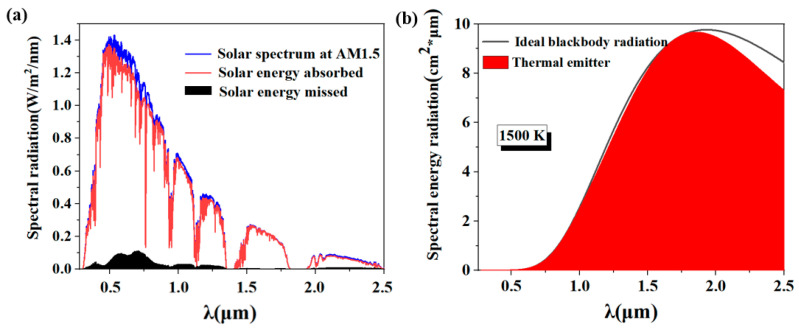
(**a**) Depicts the distribution of energy absorption and loss for solar radiation spanning from 280 nm to 2500 nm, under an atmospheric mass (AM) of 1.5; (**b**) Illustrates the energy emission spectrum emanating from a solar absorber operating at an elevated temperature of 1500 K.

Under AM 1.5 illumination conditions, as exemplified in Figure 5a, the blue curve portrays the theoretical ideal absorption spectrum, whereas the red curve represents the actual energy absorption achieved by the structure. The black area in the figure represents the main area of energy loss, mainly concentrated in the visible light band. Nevertheless, on the whole, the band absorption rate is maintained at a high level of 95.03%, while the loss rate is controlled below 5%. Further, Figure 5b divulges the thermal emission properties exhibited by the structure when subjected to a high temperature of 1500 Kelvin. The black line in the figure represents the theoretical blackbody radiation curve and serves as a benchmark, whereas the red area represents the actual thermal radiation emitted by the structure. It can be observed from the figure that within the spectral range below 1800 nanometers, the observed thermal radiation closely aligns with the theoretical blackbody radiation curve, and only slight thermal radiation loss occurs in the band above 1800 nm. However, in the whole band, the thermal radiation efficiency remains at a high level, reaching 95.96%.

### 2.4. Effect of Varying Parameters on Absorption Outcomes

To validate the optimally chosen structural parameters within this design framework, we conducted a methodical evaluation of the influence that various structural attributes exert on absorption efficiency under the premise of ensuring that other parameters remain unchanged [54,55]. Specifically, we discussed in detail the thickness H1 of top silicon dioxide (SiO_2_), the thickness H2 of rectangular titanium (Ti) and its side length D, and the period P of the whole structure. In this process, we did not take into account the parameter changes in Ti and SiO_2_, because according to the previous analysis, the adjustment of these parameters has no significant influence on absorption efficiency.

**Figure 6:**
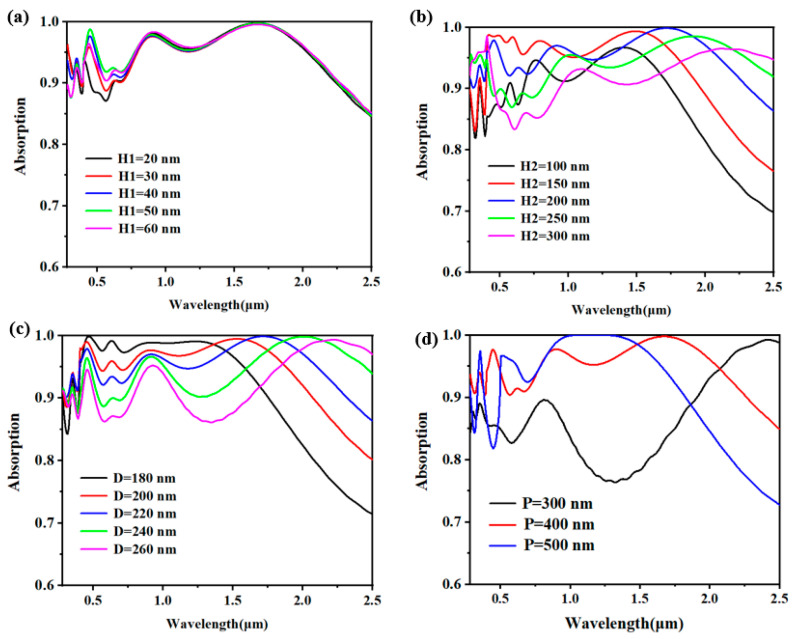
(**a**) Thickness change absorption spectrogram of top silicon dioxide; (**b**,**c**) are absorption spectrograms of thickness and side length change in rectangular titanium, respectively; (**d**) The absorption spectrum of structural period change.

In Figure 6a–d, firstly, we deeply studied how the thickness H1 of the top SiO_2_ affects the absorption spectrum. Especially in the ultraviolet and visible light bands, the SiO_2_ layer plays a vital role [56,57]. Through careful observation, we found that with the side length d gradually increasing from 20 nm to 60 nm, the absorption efficiency first showed an upward trend, but then showed a downward trend. This discovery provides an important clue for us to further understand the role of the SiO_2_ layer in light absorption. Based on this discovery, we infer that the optimal side length of a rectangle should be close to 40 nm. Then, we delved deeper into examining the effect of varying the thickness (H2) of the rectangular titanium (Ti) layer on the absorption spectrum. As H2 was incremented from 100 nm to 300 nm, we discerned a trend in the evolution of the absorption efficiency. It is particularly noteworthy that when the period p is set to 200 nm, we get the best absorption result, which not only has high absorption efficiency, but also has excellent absorption bandwidth. This discovery provides an important reference for our subsequent experiments and applications. As shown in Figure 6c, when the side length of a cuboid is increased from 180 nm to 260 nm, we can observe that the shorter the side length of the cuboid is, an enhanced absorption efficiency is observed within the short wavelength region spanning from 280 to 1500 nanometers. However, with the increase in wavelength, the absorption efficiency of the rectangular parallelepiped with longer side length is significantly enhanced and the bandwidth is wider. On the whole, when D = 220 nm, the overall absorption rate and bandwidth are better. Therefore, upon thorough analysis, we propose that a side length of D = 220 nm offers optimal performance. Figure 6d illustrates the absorption spectrum across the entire periodic variation, where it becomes evident that an increase in the periodicity corresponds to an augmentation in the absorption rate within the spectral range of 1000–1500 nanometers, but considering the overall absorption efficiency of short wave and long wave, the absorber can only exert its maximum absorption potential when P = 400 nm.

### 2.5. Angle Sensitivity Analysis

**Figure 7:**
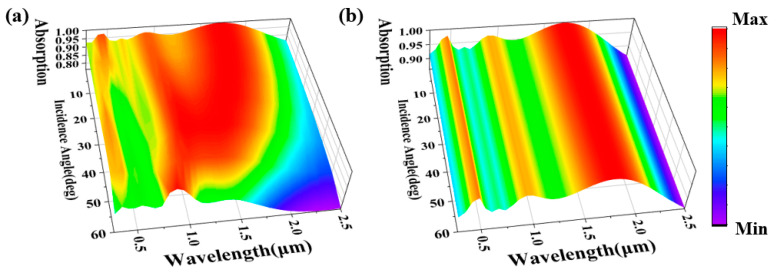
(**a**) Modifying the absorption spectrum by varying the incident angle within a range of 0° to 60°; (**b**) Manipulating the absorption spectrum through alterations in the polarization angle, spanning from 0° to 60°.

Finally, in order to study the application potential of the structure, we studied the angular sensitivity of the structure [58,59,60]. It is acknowledged that in practical scenarios, natural light seldom strikes the solar absorber vertically, contrasting with the idealized conditions. Consequently, it is crucial to investigate the influence of varying polarization and incident angles on the performance of solar absorbers. As depicted in Figure 7, we have conducted simulations to analyze the absorption spectrum, encompassing incident angles and polarization angles ranging from 0° to 60°, respectively. As evident from Figure 7a, the designed absorber demonstrates remarkable performance within an incident angle span of 0° to 60°, exhibiting exceptional overall absorption efficiency. As the polarization angle of the incident light progresses from 0° to 60°, a marginal enhancement in visible light absorption is observed, while the near-infrared region undergoes a slight attenuation, particularly from 50%. Notably, the absorber sustains an absorption rate exceeding 90% across a broad wavelength spectrum extending up to 2040 nm. The results of this study clearly show that the absorber we designed shows excellent insensitivity to incident angle. As shown in Figure 7b, the spectral response remains stable even when the polarization angle increases; this underscores the exceptional insensitivity of the structure towards polarization variations, thereby reinforcing its robustness. The inherent high geometric symmetry of the structure contributes to a consistently high absorption rate across the entire wavelength spectrum, exhibiting minimal variation with alterations in polarization angle [61,62]. This robustness to both oblique incidence and polarization insensitivity significantly enhances the absorber’s performance, presenting substantial advantages for practical applications.

## 3. Modelling and Structural Parameters of the Micro-Nano Optical Devices

**Figure 8:**
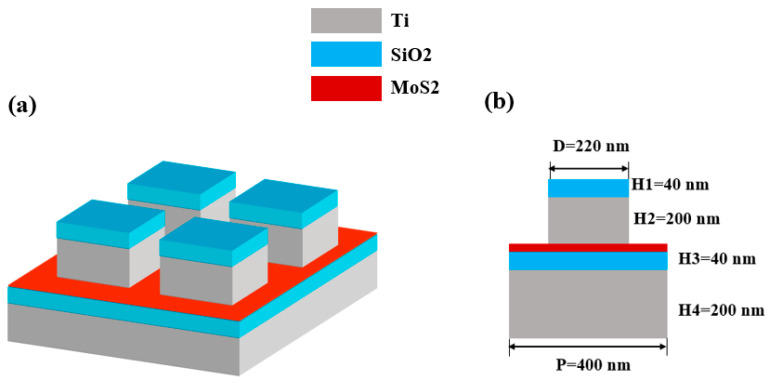
(**a**) Shows the three-dimensional structure of the model; (**b**) Structure diagram of XOZ plane.

In this paper, we introduce a periodically arranged rectangular configuration consisting of Ti-SiO_2_ layered structures, as shown in Figure 8a. The thickness of local structure is denoted as H1, H2, H3, and H4 from top to bottom. The overall width of the absorber is P = 400 nm, the bottom is made of titanium, and its thickness H4 = 300 nm. In this architecture, Ti functions as a reflective element, with its thickness (H4) set at 300 nanometers, significantly exceeding the penetration depth of electromagnetic radiation. Consequently, the transmission through this structure is effectively nullified, that is, T(ω) = 0 [63,64]. In this work, the spectral absorption efficiency is defined as:

*A*(*ω*) = *1* − *R*(*ω*) − *T*(*ω*) (3)

Therefore, the absorption rate can be simplified as *A*(*ω*) *= 1 − R*(*ω*) [65,66], where *R* (*Ω*) and *T* (*Ω*) respectively represent the spectral reflection and transmission under the illumination of plane light. The second layer is dielectric silicon dioxide (SiO_2_) with a thickness of H3 = 40 nm. In this paper, we propose a material as the supporting substrate of MoS_2_, aiming at solving the challenge of depositing MoS_2_ directly on the titanium layer. This support material is located under the dielectric material and above it is the two-dimensional metamaterial MoS_2_. We set the thickness of MoS_2_ as 0.625 nm, which is based on the standard thickness grown under most laboratory conditions, thus ensuring the accuracy and repeatability of the research. This configuration not only optimizes the growth environment of MoS_2_, but also lays a foundation for its performance in subsequent applications. The microstructure of the surface layer is stacked by Ti-SiO_2_, and its height is set to H1 = 40 nm and H2 = 200 nm. Non-precious metal (Ti) is chosen as the metal material here, because Ti has high loss in visible light and near-infrared, which can effectively broaden the absorption bandwidth, and the price is relatively cheap, which can reduce the manufacturing cost. Among them, the dielectric constant data of Ti and SiO_2_ are cited and applied based on the experimental results of Palik [67]. In numerical calculations and simulations, the dielectric constant of molybdenum disulphide (MoS_2_) is a key parameter that determines the material’s response properties to light. For the monolayer MoS_2_ in the paper, we cite the data of Ermolaev et al. [68]. Its dielectric constant is usually expressed in complex form, including real and imaginary parts, and can be expressed as n = n′ + ik, where n′ is the real part of the dielectric function, which represents the material’s polarization capacity, while k is the imaginary part, which is related to the material’s absorption capacity.

In the numerical simulation calculation, we use FDTD version 2020 solutions software as an analysis tool. In this simulation, the setting of the light source is very important. We selected the plane light source from 280 nm to 2500 nm and ensured the polarization of light aligned along the X-axis by configuring the incident light to propagate vertically in the negative Z-axis direction. To uphold the rigor and fidelity of our simulation, we applied periodic boundary conditions in the x and y dimensions to simulate the infinite extended periodic structure. Along the Z-axis, we choose the perfectly matched layer (PML) as the boundary condition to effectively absorb and eliminate the possible reflected waves during the simulation, thus ensuring the accuracy of the simulation results [69].

## 4. Conclusions

In this paper, we have achieved the successful design of an efficient broadband solar absorber, leveraging the unique properties of MoS_2_ two-dimensional materials. Through numerical calculation, our findings indicate that the absorption efficiency of the structure surpasses 90% within a wavelength spectrum exceeding 2040 nm, and the absorption rate can reach 99% or more in the wavelength range of 303 nm. It is noteworthy that an optimal thickness of 0.625 nm for the MoS_2_ layer has been identified, and its absorption bandwidth is significantly extended to 310 nm, which fully verifies the superiority of two-dimensional materials in improving absorption performance. In addition, MoS_2_ not only has excellent thermal stability, but also helps to reduce device size and improve overall absorption efficiency. Under the condition of AM 1.5 spectrum, our structure shows excellent weighted average absorption efficiency, reaching a high level of 95.03%. Even at an extreme temperature as high as 1500 K, its thermal radiation efficiency can be maintained at an excellent level of 95.96%. The structure demonstrates remarkable independence and resilience towards variations in both the polarization angle and the incident angle of the incoming light, and its performance remains stable even in the polarization angle range of 0 to 60. In summary, the high absorption efficiency and stability at extreme temperatures of this MoS_2_-based broadband solar absorber we have designed make it ideal for solar thermal photovoltaic systems that can convert solar energy directly into electricity, thereby reducing dependence on fossil fuels and lowering greenhouse gas emissions. Additionally, by using cost-effective titanium (Ti) in place of expensive precious metals, our technology helps lower the economic barrier to solar technology, advancing its use in a wider range of applications. Our design also demonstrates insensitivity to the direction of polarization of incident light, which increases its reliability in variable environments and opens up the possibility of applying solar absorbing technologies in different geographical locations and climates.

## Figures and Tables

**Table 1:** Comparison with other literature.

Essay	Spectral Region Exhibiting an Absorption Rate Exceeding 90%	Average Absorption Efficiency	Maximum Absorption Rate
[32]	712 nm	97%	99.80%
[12]	<1000 nm	85%	97.00%
[38]	1110 nm	<90%	99.80%
[34]	475 nm	94%	97%
[35]	1547 nm	90%	98%
[36]	1200 nm	91%	\
[37]	1100 nm	99.6%	98.5%
This text	2040 nm	94.61%	99.87%

## Data Availability

Publicly available datasets were analyzed in this study. These data can be found here: [https://www.lumerical.com/] (accessed on 1 January 2020).
